# Viral Inactivation with Emphasis on SARS-CoV-2 Using Physical and Chemical Disinfectants

**DOI:** 10.1155/2021/9342748

**Published:** 2021-10-25

**Authors:** Abdolmajid Fadaei

**Affiliations:** Department of Environmental Health Engineering, School of Health, Shahrekord University of Medical Sciences, Shahrekord, Iran

## Abstract

**Background:**

Recently, an outbreak of a novel human coronavirus SARS-CoV-2 has become a world health concern leading to severe respiratory tract infections in humans. Virus transmission occurs through person-to-person contact, respiratory droplets, and contaminated hands or surfaces. Accordingly, we aim at reviewing the literature on all information available about the persistence of coronaviruses, including human and animal coronaviruses, on inanimate surfaces and inactivation strategies with biocides employed for chemical and physical disinfection.

**Method:**

A comprehensive search was systematically conducted in main databases from 1998 to 2020 to identify various viral disinfectants associated with HCoV and methods for control and prevention of this newly emerged virus.

**Results:**

The analysis of 62 studies shows that human coronaviruses such as severe acute respiratory syndrome (SARS) coronavirus, Middle East respiratory syndrome (MERS) coronavirus or endemic human coronaviruses (HCoV), canine coronavirus (CCV), transmissible gastroenteritis virus (TGEV), and mouse hepatitis virus (MHV) can be efficiently inactivated by physical and chemical disinfectants at different concentrations (70, 80, 85, and 95%) of 2-propanol (70 and 80%) in less than or equal to 60 s and 0.5% hydrogen peroxide or 0.1% sodium hypochlorite within 1 minute. Additionally, glutaraldehyde (0.5–2%), formaldehyde (0.7–1%), and povidone-iodine (0.1–0.75%) could readily inactivate coronaviruses. Moreover, dry heat at 56°C, ultraviolet light dose of 0.2 to 140 J/cm^2^, and gamma irradiation could effectively inactivate coronavirus. The WHO recommends the use of 0.1% sodium hypochlorite solution or an ethanol-based disinfectant with an ethanol concentration between 62% and 71%.

**Conclusion:**

The results of the present study can help researchers, policymakers, health decision makers, and people perceive and take the correct measures to control and prevent further transmission of COVID-19. Prevention and decontamination will be the main ways to stop the ongoing outbreak of COVID-19.

## 1. Introduction

Like other countries of the world, Iran has been adversely affected by the pandemic of coronavirus disease 2019 (COVID-19) caused by severe acute respiratory syndrome coronavirus 2 (SARS-CoV-2) and Middle East respiratory syndrome (MERS) coronavirus. As the third highly pathogenic human coronavirus, COVID-19 has emerged in the last two decades. The virus transmission through human-to-human contact has been reported both in hospital and family settings [1].

Since the 1970s, approximately 40 novel infectious diseases have been detected, and over the last 20 years, several major outbreaks have occurred, including SARS (2002-2003), H1N1 flu (swine flu) (2009-2010), Ebola (2014–2016), Zika virus (2015-2016), and COVID-19 (2019-2020) [2, 3]. Though respiratory pathogens, such as flu, spread through airborne dispersion of small particle aerosols (≤5 *μ*m) generated by breathing, coughing, or sneezing of an infected person, respiratory syncytial viruses, SARS-CoV, and MERS-CoV can be transmitted via large droplets blown through the air into the eyes, nose, and mouth in a short distance [4, 5]. A variety of disinfectants such as sodium hypochlorite, hydrogen peroxide, alcohols, povidone-iodine, or glutardialdehyde, ultraviolet radiation, and dry heat or moist heat are used across the world for disinfection, mainly in hospitals [6, 7].

By this review, we aimed to provide a summary of all information available on the persistence of all coronaviruses, including emerging SARS-CoV and MERS-CoV, as well as bestial coronaviruses such as contagious canine coronavirus (CCV), transmissible gastroenteritis virus (TGEV), and mouse hepatitis virus (MHV), on various types of inanimate surfaces and the efficacy of the widely applied surface disinfectants used against different types of viruses and coronaviruses.

## 2. Method

**Figure 1:**
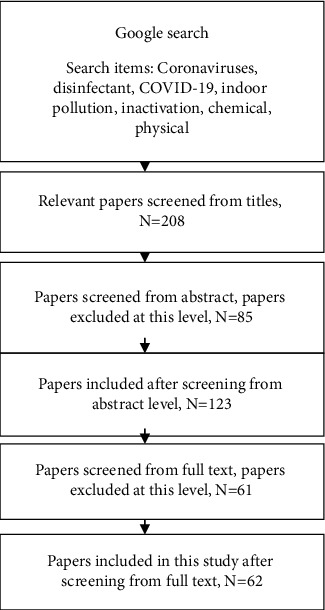
Schematic diagram of the analysis process.

In order to identify various viral disinfectants associated with HCoV and methods for control and prevention of this newly emerged virus, a comprehensive search was systematically conducted between 1998 and 2020 in main databases, including the Elsevier Bibliographic Database (Scopus), Institute for Scientific Information (ISI) Web of Science, Google Scholar, and PubMed (MEDLINE), using free text words, MeSH (Medical Subject Headings), and their possible combination. The last search was conducted on November 25, 2020. We searched in the aforementioned databases with proper keywords: (“Viruses” OR “Coronaviruses” OR “CoV” OR “Human Coronaviruses “OR “HCoV” OR “nCov” OR “Novel Coronaviruses” OR “2019 Novel Coronavirus” OR “Covid-19” OR “2019-nCoV” OR “Severe Acute Respiratory Syndrome- Coronaviruses-2” OR “SARS-COV-2”) AND (“Disinfectant” OR “Disinfection” OR “Chemical Inactivation” OR “Physical Inactivation” OR “Biocidal Agents”). To avoid bias, we systematically investigated the title, abstract, and full text of the studies. Then, information such as first author's name, publication year, country, type of disinfectant (a few disinfectants used the commercial brand), concentration, type of virus, strain/isolate, contact time, reduction of viral infectivity (log_10_), and main findings was extracted. A total of 208 peer-reviewed publications were accessed based on the relevance of titles to the research. These were further screened to 123 after reading through their abstracts. After screening the full text of the papers, 62 cases were used for this review, excluding the Preferred Reporting Items for Systematic Reviews and Meta-Analyses (PRISMA) reference [8]. Papers were excluded as a result of irrelevant abstracts to our review goals, inconsistent titles with abstract and full-text content, insufficient presentation of information, use of irrelevant statistical tools, not supported scientific claims, or extreme violation of certain assumptions in results' explanation and discussion (Figure 1).

## 3. Results

According to the searched studies, different types of physical disinfectants used for viral inactivation include dry heat, moist heat, gamma irradiation, visible light plus methylene blue (MB) (plasma units), and ultraviolet C (Table 1). Moreover, among chemical disinfectants, ethanol (70–95%), 2-propanol (70–100%), the combination of 45% 2-propanol with 30% 1-propanol, and Desderman (78% ethanol) readily inactivated coronavirus, poliovirus, and adenovirus infectivity by approximately 2–5 log_10_ or more. Hydrogen peroxide was effective at a concentration of 0.5% and a contact time of 1 min with virucidal efficacy against coronavirus and influenza type A. Sodium hypochlorite (0.001–0.6%) readily inactivated poliovirus, adenovirus, Ebola, HIV, and coronavirus infectivity by approximately 0.9–7 log_10_ or more. Glutaraldehyde (0.5–2%), formaldehyde (0.7–1%), and povidone-iodine (0.1–0.75%) could readily inactivate different viruses including type A influenza virus, a type of coronavirus (Table 2).

## 4. Discussion

Viral inactivation by physical and chemical disinfectants has a wide application in human disease-control programs to prevent the spread of viral infectious diseases. The veridical activity of chemical compounds and physical agents cannot be predicted reliably only on the basis of their mechanism of action and the nature and morphology of the viruses to be inactivated. Physical disinfectants including UVC disinfection system for surface disinfection in 11–34 min exposure time with more than (5 log_10_ reduction) 99.999% efficiency can inactivate SARS-CoV-2. Additionally, UVC light affects viruses, such as vaccinia virus, CCHFV, and NIV with inactivation efficiency more than 99% (2-log_10_ reduction). One study reported higher susceptibility of nonenveloped viruses to UVC radiation than enveloped ones [31]. Exposure to UV-C radiation showed no significant effects on canine coronavirus inactivation for up to 3 days [22]. Findings of one study showed that SARS-CoV-2 is 3-fold more sensitive to UV than influenza [10]. Another study showed that ultraviolet light at 134 W/cm^2^ for 15 min inactivates the infectivity of SARS-CoV-2 by 99.999% [11]. Another study showed that ultraviolet light at 0.162 W/cm^2^ for 30 min inactivates infectivity of SARS-CoV and provides less than 1-log_10_ reduction [32]. Ultraviolet radiation in sunlight is the primary virucidal agent in the environment [11]. Until now, most useful cases of coronavirus inactivation have been accomplished using mercury vapor lamps with peak radiation at 254 nm [33]. Gamma irradiation at 1 mrad is effective for the inactivation of coronavirus in a short contact time. Dry heat (56°C, 60°C, 80°C, and 90°C) is used to inactivate poliovirus, adenovirus, and a type of coronavirus, and moist heat is used to inactivate poliovirus and adenovirus. One study reported that CCV was completely inactivated at 65°C after 40 min and at 75°C after 30 min [22, 34]. Another study found that, at 56°C, most of the viruses (SARS-CoV-2) were inactivated after 20 min [35]. One study reported that SARS-CoV was completely inactivated at 56°C after 90 min, at 67°C after 60 min, and at 75°C after 30 min [32]. Dry heat at 60°C for 30 min, 65°C for 15 min, and 80°C for 1 min could vigorously decrease coronavirus infectivity by at least 99.99% [36]. One study reported that SARS-CoV-2 was completely inactivated at 56°C after 30 min, at 60°C after 60 min, and at 92°C after 15 min [37].

### 4.1. Inactivation of Viruses by Chemical Disinfectants

Various chemical disinfectant compounds such as Desderman (78% ethanol), ethanol with different concentrations (70, 80, 85, and 95%), 2-propanol (concentration of 70 and 80%), and Sterillium (45% 2-propanol and 30% 1-propanol) are used for viral inactivation. The WHO has recommended the use of alcohol-based (containing at least 60% alcohol) hand sanitizers when soap and water are not available [38]. Ethanol 62–71% used for disinfection of small surfaces exhibited a similar efficacy against coronavirus [39].

Hydrogen peroxide vapor in the gas phase at a concentration of 0.05% required 1 min exposure time to inactivate the influenza type A virus—a type of coronavirus. A minimum concentration of sodium hypochlorite of 0.001% could effectively inactivate some types of viruses in 1 min. On the contrary, sodium hypochlorite 0.6% required more contact time to inactivate poliovirus, adenovirus, Ebola, HIV, and coronavirus. According to our data in this study, the WHO also recommended the use of bleach solution at concentrations of 1 to 5% for disinfection of surfaces at different times [40]. Bleach is typically used at a concentration of 0.05% [39]. Authors found that sodium hypochlorite 6% and phenolic compounds 5% were effective in inactivation of coronavirus [41]. Disinfectants such as formaldehyde (0.7–1%) and glutaraldehyde (0.5–2%) were effective in inactivation of coronavirus. Glucoprotamin (26%) and magnesium monoperphthalate (0.5%) also required more contact time for the destruction of coronavirus. One study reported that CCV was incubated with formaldehyde (0.036% and 0.009%) and glutaraldehyde (0.002% and 0.001%) at different temperatures to evaluate their virus-inactivating potential [41]. Glutaraldehyde is a main dialdehyde used as a disinfectant and sterilizer, specifically for hospital instruments, and is widely applicable as a means of inactivating viruses, bacteria and their spores, and fungi [22]. Povidone-iodine with a concentration of 0.1, 0.23, 0.4, and 0.75% could readily inactivate different coronaviruses and influenza type A infectivity in 15 s by approximately more and equal to 4-log_10_ (≥99.99). Another study showed that UVC irradiation, dry heat, formaldehyde (formalin), glutaraldehyde, and excessive pH value were able to inactivate SARS-CoV [35]. The enveloped viruses such as coronavirus and influenza were more readily inactivated by physical and chemical disinfectants than nonenveloped viruses, such as adenovirus and poliovirus.

### 4.2. General Recommendations to Be Followed

Before using any disinfectant, any surface should be initially cleaned with a water and detergent solution. Then, healthcare disinfectants such as formaldehyde, glutaraldehyde, and sodium hypochlorite can be applied.

The disinfectant concentration and exposure time play the most important role in surface viral inactivation.

## 5. Conclusions

In conclusion, the obtained results indicated that the WHO-recommended alcohol-based formulations were validated with various enveloped viruses. A strong veridical effect against newly emerged pathogens, including EBOV, SARS-CoV, and MERS-CoV, could be demonstrated implicating the applicability of these WHO formulations in healthcare, public health, and outbreak associated with these types of viruses.

## Figures and Tables

**Table 1:** Inactivation of viruses by different types of physical disinfectants.

Disinfection	Concentration	Virus	Strain isolate	Retention time	Reduction of viral infectivity (log_10_)	Reference
UVC (254 nm)	Dose of 3 J/m^2^	SARS-CoV-2	Isolate FFM-1	11–34 min	1.0	[9]
UVC (254 nm)	Dose of 7 J/m^2^	SARS-CoV-2	Isolate FFM-1	11–34 min	2.0	[9]
UVC (254 nm)	Dose of 28 J/m^2^	SARS-CoV-2	Isolate FFM-1	11–34 min	3.0	[9]
UVC (254 nm)	Dose of 140 J/m^2^	SARS-CoV-2	Isolate FFM-1	11–34 min	6.0	[9]
UVC (254 nm)	Dose of 6.9 J/m^2^	SARS-CoV-2	—	2 h	1.0	[10]
UVB (300 nm)	Dose of 1400 J/m^2^	Vaccinia virus	—	2 h	2.0	[11]
UVC (254 nm)	Dose of 0.2 J/cm^2^	SARS-CoV-2	Frankfurt 1	—	≥3.4	[12]
UVC (254 nm)	Dose of 0.2 J/cm^2^	CCHFV	Afg09-2990	—	≥2.2	[12]
UVC (254 nm)	Dose of 0.2 J/cm^2^	NiV	Malaysia	—	≥4.3	[12]
UVC (222 nm)	Dose of 0.56 mJ/cm^2^	HCoV	229E	—	1.0	[13]
UVC (222 nm)	Dose of 0.39 mJ/cm^2^	HCoV	OC43	—	1.0	[13]
Pulsed-xenon ultraviolet light	—	SARS-CoV-2	USA-WA1/2020	2 min	>4.54	[14]
Visible light plus MB (plasma units)	120 J/cm^2^	MERS-CoV	EMC/2012	—	≥3.3	[12]
Visible light plus MB (plasma units)	120 J/cm^2^	EBOV	Mayinga-76	—	≥4.6	[12]
Gamma irradiation	1 mrad	SARS-CoV-1	Strain Tor 2	5 s	>4.0	[15]
Dry heat	90°C	Poliovirus	Strain Sabin	1 min	>4.6	[16]
Dry heat	90°C	Adenovirus	Type 5	1 min	3.9	[16]
Dry heat	60°C	CCV	SP-80 strain	1 min	1.56	[17]
Dry heat	60°C	MHV	RV-13 strain	1 min	2.87	[17]
Dry heat	80°C	CCV	SP-80 strain	1 min	>4.04	[17]
Dry heat	80°C	MHV	RV-13 strain	1 min	>3.88	[17]
Dry heat	56°C	SARS-CoV	Isolate FFM-1	30 min	≥5.01	[18]
Dry heat	60°C	SARS-CoV	Isolate FFM-1	30 min	≥5.01	[18]
Dry heat	4°C	PEDV	Strain CV777	120 min	0.0	[19]
Dry heat	40°C	PEDV	Strain CV777	120 min	1.0	[19]
Dry heat	56°C	PEDV	Strain CV777	10 min	1.0–1.7	[20]
Dry heat	56°C	MERS-CoV	MERS-CoV strain Hu/France-FRA2_130569/2013 (FRA2)	30 s	0.1–0.9	[21]
Dry heat	56°C	CCV	Strain S378	30 min	≥5.01	[22]
Moist heat	90°C	Poliovirus	Strain Sabin	1 min	>5.0	[18]
Moist heat	90°C	Adenovirus	Type 5	1 min	>4.1	[18]

CCHFV: Crimean-Congo haemorrhagic fever virus; NiV: Nipah virus; EBOV: Ebola virus; MB: methylene blue; SARS: severe acute respiratory syndrome; MERS: Middle East respiratory syndrome; MHV: mouse hepatitis virus; CCV: canine coronavirus; HCoV: human coronavirus; TGEV: transmissible gastroenteritis virus; PEDV: porcine epidemic diarrhoea virus.

**Table 2:** Inactivation of viruses by different types of chemical disinfectants.

Disinfection	Concentration	Virus	Strain isolate	Contact time	Reduction of viral infectivity (log_10_)	Reference
Ethanol	95%	SARS-CoV	Isolate FFM-1	30 s	≥5.5	[23]
Ethanol	80%	MERS-CoV	Strain EMC	30 s	>4.0	[24]
Ethanol	70%	Poliovirus	Strain Sabin	1 min	2.1	[16]
Ethanol	70%	Adenovirus	Type 5	1 min	2.4	[16]
2-Propanol	100%	SARS-CoV-2	Isolate FFM-1	30 s	≥3.31	[23]
2-Propanol	70%	SARS-CoV-2	Isolate FFM-1	30 s	≥3.31	[23]
Desderman (78% ethanol)	78%	SARS-CoV-2	Isolate FFM-1	30 s	≥5.01	[23]
Sterillium (45% 2-propanol and 30% 1-propanol)	45% and 30%	SARS-CoV-2	Isolate FFM-1	30 s	≥2.78	[23]
Hydrogen peroxide	Vapor of unknown concentration	TGEV	Purdue strain type 1	2-3 h	4.9–5.3	[25]
Hydrogen peroxide	0.5%	HCoV	Strain 229E	1 min	>4.0	[26]
Hydrogen peroxide	0.5%	Influenza A virus	PR-8	1 min	>4.75	[26]
Hydrogen peroxide	0.5%	HCoV	Type 37	1 min	>4.25	[26]
Benzalkonium chloride	0.04%	HCoV	Strain 229E	1 min	<3.0	[27]
Didecyldimethylammonium chloride	0.0025%	CCV	Strain S378	3 d	>4.0	[28]
Sodium hypochlorite	2500 ppm	Poliovirus	Strain Sabin	10 min	4.5	[16]
Sodium hypochlorite	2500 ppm	Adenovirus	Type 5	10 min	ND	[16]
Sodium hypochlorite	10 ppm	CCV	SP-80 strain	10 min	0.9	[17]
Sodium hypochlorite	100 ppm	CCV	SP-80 strain	10 min	1.05	[17]
Sodium hypochlorite	0.5–0.6%	Ebola virus	—	10 min	1.1	[29]
Sodium hypochlorite	0.5%	HIV	—	1 min	≥7	[29]
Formaldehyde	0.7%	SARS-CoV	Isolate FFM-1	2 min	>3.01	[18]
Formaldehyde	1%	SARS-CoV	Isolate FFM-1	2 min	>3.01	[18]
Glutaraldehyde	0.5%	SARS-CoV	Isolate FFM-1	2 min	>4.01	[18]
Glutaraldehyde	2%	HCoV	Strain 229E	1 min	>3.0	[27]
Glucoprotamin	26%	SARS-CoV	Isolate FFM-1	2 min	>1.68	[27]
Magnesium monoperphthalate	0.5%	SARS-CoV	Isolate FFM-1	30 min	>4.5	[27]
PVP-I surgical scrub (7.5 g/L available iodine)	7.5 g/L	MERS-CoV	Isolate HCoV-EMC/2012	15 s	4.64	[30]
PVP-I skin cleanser (4 g/L available iodine)	4 g/L	MERS-CoV	Isolate HCoV-EMC/2012	15 s	4.97	[30]
PVP-I gargle and mouthwash (1 g/L available iodine)	1 g/L	MERS-CoV	Isolate HCoV-EMC/2012	15 s	4.30	[30]
Povidone-iodine	0.23%	SARS-CoV	Isolate FFM-1	15 s	4.60	[4]
Povidone-iodine	0.23%	MERS-CoV	Isolate HCoV-EMC/2012	15 s	4.40	[4]
Povidone-iodine	0.23%	Influenza virus A	H1N1	15 s	5.67	[4]

ND: not done; SARS: severe acute respiratory syndrome; MERS: Middle East respiratory syndrome; MHV: mouse hepatitis virus; CCV: canine coronavirus; HCoV: human coronavirus; TGEV: transmissible gastroenteritis virus.

## Data Availability

All the data generated or analyzed during this study are included within the article.

## References

[1] Chan J. F.-W., Yuan S., Kok K.-H. (2020). A familial cluster of pneumonia associated with the 2019 novel coronavirus indicating person-to-person transmission: a study of a family cluster. *The Lancet*.

[2] Sims N., Kasprzyk-Hordern B. (2020). Future perspectives of wastewater-based epidemiology: monitoring infectious disease spread and resistance to the community level. *Environment International*.

[3] Acter T., Uddin N., Das J., Akhter A., Choudhury T. R., Kim S. (2020). Evolution of severe acute respiratory syndrome coronavirus 2 (SARS-CoV-2) as coronavirus disease 2019 (COVID-19) pandemic: a global health emergency. *The Science of the Total Environment*.

[4] Eggers M., Koburger-Janssen T., Eickmann M., Zorn J. (2018). In vitro bactericidal and virucidal efficacy of povidone-iodine gargle/mouthwash against respiratory and oral tract pathogens. *Infectious diseases and therapy*.

[5] Ahmadi D., Fadaei A. (2021). Efficiency evaluation of hospitals sterilization by biological and chemical methods. *Quality of Life*.

[6] Sadeghi M., Fadaei A., Ataee M. (2020). Assessment of hospitals medical waste management in Chaharmahal and Bakhtiari Province in Iran. *Archives of Agriculture and Environmental Science*.

[7] Fadaei A. (2021). Ventilation systems and COVID-19 spread: evidence from a systematic review study. *European Journal of Sustainable Development Research*.

[8] Moher D., Liberati A., Tetzlaff J., Altman D. G. (2010). Preferred reporting items for systematic reviews and meta-analyses: the PRISMA statement. *International Journal of Surgery*.

[9] Lytle C. D., Sagripanti J.-L. (2005). Predicted inactivation of viruses of relevance to biodefense by solar radiation. *Journal of Virology*.

[10] Sagripanti J. L., Lytle C. D. (2020). Estimated inactivation of coronaviruses by solar radiation with special reference to COVID-19. *Photochemistry and Photobiology*.

[11] Sagripanti J.-L., Voss L., Marschall H.-J., David Lytle C. (2013). Inactivation of vaccinia virus by natural sunlight and by artificial UVB radiation. *Photochemistry and Photobiology*.

[12] Eickmann M., Gravemann U., Handke W. (2020). Inactivation of three emerging viruses severe acute respiratory syndrome coronavirus, Crimean-Congo haemorrhagic fever virus and Nipah virus-in platelet concentrates by ultraviolet C light and in plasma by methylene blue plus visible light. *Vox Sanguinis*.

[13] Manuela B., Welch D., Igor S., Brenner D. J. (2020). Far-UVC light (222 nm) efficiently and safely inactivates airborne human coronaviruses. *Scientific Reports*.

[14] Simmons S. E., Carrion R., Alfson K. J. (2020). Deactivation of SARS-CoV-2 with pulsed-xenon ultraviolet light: implications for environmental COVID-19 control. *Infection Control & Hospital Epidemiology*.

[15] Feldmann F., Shupert W. L., Haddock E., Twardoski B., Feldmann H. (2019). Gamma irradiation as an effective method for inactivation of emerging viral pathogens. *The American Journal of Tropical Medicine and Hygiene*.

[16] Eterpi M., McDonnell G., Thomas V. (2009). Disinfection efficacy against parvoviruses compared with reference viruses. *Journal of Hospital Infection*.

[17] Saknimit M., Inatsuki I., Sugiyama Y., Yagami K.-I. (1988). Virucidal efficacy of physico-chemical treatments against coronaviruses and parvoviruses of laboratory animals. *Experimental Animals*.

[18] Rabenau H. F., Cinatl J., Morgenstern B., Bauer G., Preiser W., Doerr H. W. (2005). Stability and inactivation of SARS coronavirus. *Medical Microbiology and Immunology*.

[19] Quist-Rybachuk G., Nauwynck H., Kalmar I. (2015). Sensitivity of porcine epidemic diarrhea virus (PEDV) to pH and heat treatment in the presence or absence of porcine plasma. *Veterinary Microbiology*.

[20] Hulst M. M., Heres L., Honing R. W., Pelser M., Fox M., Poel W. H. M. (2019). Study on inactivation of porcine epidemic diarrhoea virus, porcine sapelovirus 1 and adenovirus in the production and storage of laboratory spray‐dried porcine plasma. *Journal of Applied Microbiology*.

[21] Leclercq I., Batéjat C., Burguière A. M., Manuguerra J. C. (2014). Heat inactivation of the M iddle E ast respiratory syndrome coronavirus. *Influenza and other respiratory viruses*.

[22] Pratelli A. (2008). Canine coronavirus inactivation with physical and chemical agents. *The Veterinary Journal*.

[23] Rabenau H. F., Kampf G., Cinatl J., Doerr H. W. (2005). Efficacy of various disinfectants against SARS coronavirus. *Journal of Hospital Infection*.

[24] Siddharta A., Pfaender S., Vielle N. J. (2017). Virucidal activity of world health organization-recommended formulations against enveloped viruses, including Zika, Ebola, and emerging coronaviruses. *Journal of Infectious Diseases*.

[25] Goyal S. M., Chander Y., Yezli S., Otter J. A. (2014). Evaluating the virucidal efficacy of hydrogen peroxide vapour. *Journal of Hospital Infection*.

[26] Omidbakhsh N., Sattar S. A. (2006). Broad-spectrum microbicidal activity, toxicologic assessment, and materials compatibility of a new generation of accelerated hydrogen peroxide-based environmental surface disinfectant. *American Journal of Infection Control*.

[27] Kampf G., Todt D., Pfaender S., Steinmann E. (2020). Persistence of coronaviruses on inanimate surfaces and their inactivation with biocidal agents. *Journal of Hospital Infection*.

[28] Pratelli A. (2007). Action of disinfectants on canine coronavirus replication in vitro. *Zoonoses and public health*.

[29] Rutala W. A., Weber D. J. (1997). Uses of inorganic hypochlorite (bleach) in health-care facilities. *Clinical Microbiology Reviews*.

[30] Eggers M., Eickmann M., Zorn J. (2015). Rapid and effective virucidal activity of povidone-iodine products against Middle East respiratory syndrome coronavirus (MERS-CoV) and modified vaccinia virus Ankara (MVA). *Infectious diseases and therapy*.

[31] Kweinor Tetteh E., Opoku Amankwa M., Armah E. K., Rathilal S. (2020). Fate of COVID-19 occurrences in wastewater systems: emerging detection and treatment technologies-a review. *Water*.

[32] Duan S.-M., Zhao X.-S., Wen R.-F. (2003). Stability of SARS coronavirus in human specimens and environment and its sensitivity to heating and uv irradiation. *Biomedical and Environmental Sciences*.

[33] Heßling M., Hönes K., Vatter P., Lingenfelder C. (2020). Ultraviolet irradiation doses for coronavirus inactivation–review and analysis of coronavirus photoinactivation studies. *GMS Hygiene and Infection Control*.

[34] Fadaei A. (2014). Comparison of environmental health indices of private clinics in Chramahal and Bakhtiari province, Iran. *Advances in Environmental Biology*.

[35] Darnell M. E. R., Subbarao K., Feinstone S. M., Taylor D. R. (2004). Inactivation of the coronavirus that induces severe acute respiratory syndrome, SARS-CoV. *Journal of Virological Methods*.

[36] Kampf G., Voss A., Scheithauer S. (2020). Inactivation of coronaviruses by heat. *Journal of Hospital Infection*.

[37] Pastorino B., Touret F., Gilles M., de Lamballerie X., Charrel R. N. (2020). Heat inactivation of different types of SARS-CoV-2 samples: what protocols for biosafety, molecular detection and serological diagnostics?. *Viruses*.

[38] United States Environmental Protection Agency (2020). *Workplaces B: Guidance For Cleaning And Disinfecting*.

[39] WHO (2014). *Infection Prevention and Control of Epidemic-And Pandemic-Prone Acute Respiratory Infections in Health Care*.

[40] Marnie C., Peters M. (2020). *ANMF Evidence Brief Covid-19: Cleaning and Disinfection of Hospital Surfaces and Equipment*.

[41] Protano C., Vitali M., Raitano A., Sancin A., Agolini G. (2008). Is there still space for the implementation of antisepsis and disinfection to prevent rotavirus and norovirus gastroenteritis outbreaks?. *Journal of preventive medicine and hygiene*.

